# Modelling Purcell enhancement of metasurfaces supporting *quasi*-bound states in the continuum

**DOI:** 10.1515/nanoph-2025-0456

**Published:** 2025-12-09

**Authors:** Joshua T. Y. Tse, Taisuke Enomoto, Shunsuke Murai, Katsuhisa Tanaka

**Affiliations:** Department of Material Chemistry, Graduate School of Engineering, Kyoto University, Katsura, Kyoto, 615-8510, Japan; Department of Physics and Electronics, Graduate School of Engineering, 12918 Osaka Metropolitan University, Osaka, 599-8531, Japan

**Keywords:** bound states in the continuum, coupled-mode theory, metasurfaces, Purcell effect

## Abstract

Bound states in the continuum (BIC) exhibit extremely high quality factors due to the lack of radiation loss and thus are widely studied for Purcell enhancement. However, a closer examination reveals that the enhancement is absent at the BIC due to the lack of out-coupling capability, but the strong enhancement is only observed at nearby configuration, namely *quasi*-BIC. To study this unique behavior of the Purcell enhancement near BIC, we built an analytical model with spectral parameters to analyze the Purcell enhancement on metasurfaces supporting *quasi*-BIC. Our analytical model predicts the average Purcell enhancement by metasurfaces coupled to a luminescent medium, utilizing parameters that are formulated through the temporal coupled-mode theory and can be derived from measured spectra such as transmissivity and reflectivity. We analyzed several metasurfaces supporting *quasi*-BIC numerically and experimentally to study the behavior of the spectral parameters as well as the resultant Purcell enhancement. We formulated the interdependence between the quality factor and the out-coupling efficiency, and revealed the existence of optimal detuning from the BIC. We also discovered that our findings are general and applicable towards realistic metasurfaces that are lossy and/or asymmetric. This discovery provides an intuitive model to understand the modal qualities of *quasi*-BIC and will facilitate optimization of *quasi*-BIC for luminescence enhancement applications.

## 1 Introduction

Bound states in the continuum (BIC) is a concept that was first proposed in quantum mechanics, but eventually rose as a general wave phenomenon that has developed significant influence in numerous fields of physics [1], [2], [3], [4], [5]. In nanophotonics, BIC describe localized optical modes that share the same energy and momentum with planewaves (the continuum), but yet are incapable of coupling with the continuum, thus being a bound state [6], [7], [8]. There are two main types of BIC: symmetry-protected and accidental. Symmetry-protected BIC are prevented from coupling with planewaves due to symmetry mismatch between the localized optical modes and the planewave, while accidental BIC occur through Friedrich–Wintgen interference, where destructive interference between multiple leaky resonances cancels out each other and results in trapped states [2], [8], [9], [10]. Due to the bound state nature, BIC exhibit diverging radiative quality (*Q*) factors, which theoretically can lead to an infinite *Q* factor in an ideal system. In applications, BIC are often intentionally detuned to achieve weakly radiative modes with similarly high *Q* factors, namely *quasi*-BIC (*q*-BIC) [11], [12], [13], [14], [15], [16].

Photoluminescence on metasurfaces [17], [18], [19], nanoplasmonics [20], [21], and photonic crystals [22], [23], [24] supporting BIC have been studied extensively due to the extraordinarily high *Q* factor. The Purcell effect describes the enhancement in spontaneous emission rate of quantum emitters when placed in an optical cavity [25], [26], [27]. The increase in local density of optical states (LDOS) enhances the spontaneous emission rate, and the maximum enhancement is given by the Purcell factor $P_{f}=\frac{3}{4\pi^{2}}{\left(\frac{\lambda }{n}\right)}^{3}\frac{Q}{V}$ , where *V* is the effective modal volume [25]. As we look closer to the Purcell enhancement of *q*-BIC, we observe the unique feature that is the absence of any enhancement precisely at the BIC, even with precisely fabricated and high quality systems [16], [17], [28]. Intuitively, we understand this is due to the lack of out-coupling channels available for the emission as the detuning from the BIC diminishes. However, a comprehensive theory that accurately describes how this lack of out-coupling channels interacts with other factors that compose the Purcell factor, and ultimately leads to the absence of enhancement at the BIC, is still missing.

**Figure 1:**
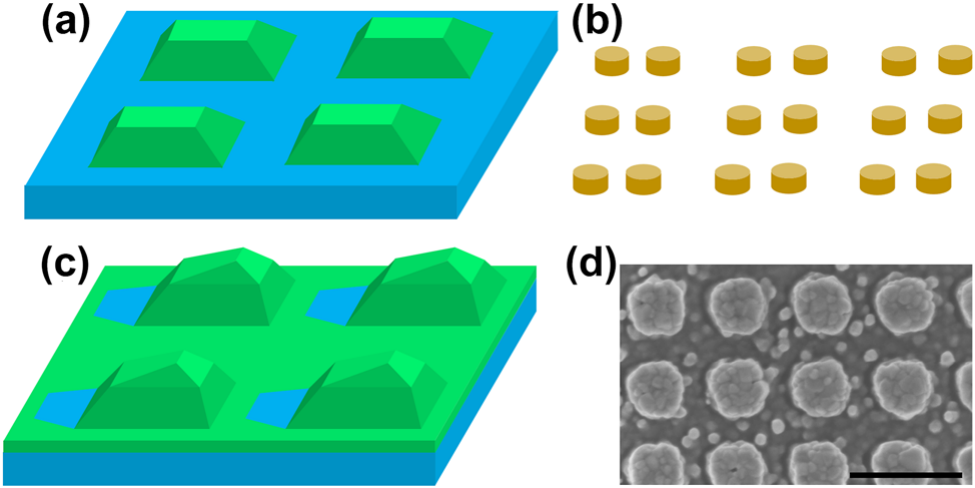
The metasurfaces that support *q*-BIC are illustrated. The numerically simulated (a) lossless TiO_2_ metasurface, (b) lossy bipartite Si metasurface, (c) asymmetric TiO_2_ metasurface and (d) experimental asymmetric TiO_2_ metasurface are investigated in Section 3.1–3.4. The colors used are: TiO_2_: green, SiO_2_ (substrate): blue, Si: orange. The scale bar in the SEM image is 500 nm.

Recently, we derived an analytical model that uses spectral parameters to predict the average Purcell enhancement and examined the photoluminescence enhancement (PLE) in systems that support surface lattice resonance (SLR) [29]. We discovered the spectral parameter that correlates with the nearfield confinement, which enables us to predict the Purcell enhancement while circumventing the need for measuring and integrating the nearfield. This ensures that our analytical model is compatible with experimental scenarios, where spectral measurements are reasonably accessible, but the nearfield is not. We also revealed that the three contributing factors to the measurable PLE are the *Q* factor, the nearfield confinement, and the out-coupling efficiency, with the *Q* factor being the most influential factor in SLR. Here, we expand upon this analytical model to analyze the influence of the out-coupling channels on the PLE of *q*-BIC. We first derive the equation that predicts the PLE of *q*-BIC with spectral parameters, in which we highlight the importance of the out-coupling efficiency for compatibility with *q*-BIC. The spectral parameters are defined based on the temporal coupled-mode theory (CMT) formalism, and the values are determined through fitting with measured or simulated spectra [30], [31], [32]. We strategically examine several metasurfaces outlined in Figure 1 that support *q*-BIC through numerical and experimental analysis to gain a deeper understanding into the PLE. We first investigate a lossless scenario that resembles most closely an ideal symmetry-protected BIC and examine the basic spectral behaviors of *q*-BIC. Then, we step-by-step introduce material loss and random scattering loss into the analysis and discuss the effect of these realistic imperfections on Purcell enhancement. Finally, we also discuss the effects of asymmetry on the spectral properties and PLE of *q*-BIC through systems that support accidental *q*-BIC.

## 2 Analytical modelling of Purcell enhancement of *q*-BIC

Numerous studies have shown that the enhancement in fractional LDOS can be predicted through the nearfield enhancement from a reciprocal source [29], [33], [34], [35], [36], [37]. If we compare the emission that leaves the metasurface and propagates away as a planewave with momentum −*ℏ* **k** (and polarization **q**), the fractional LDOS enhancement is proportional to the electric field intensity ${\left|E_{k}\left(r,\omega \right)\right|}^{2}$ under the incident *ℏ* **k** (and polarization **q**), integrated over the space occupied by the emitters. Therefore, we can obtain the PLE for a specific emission angle by comparing the nearfield with a reference:

(1) $$\text{PL}{\text{E}}_{-k}\left(\omega \right)=\frac{\int_{\text{dye}}{\left|E_{k}\left(r,\omega \right)\right|}^{2}{\text{d}}^{3}r}{\int_{\text{dye}}{\left|E_{ref,k}\left(r,\omega \right)\right|}^{2}{\text{d}}^{3}r}$$

where the ${\left|E_{k}\right|}^{2}$ of the resonance inside the dye medium under the incident *ℏ* **k** is compared to that of a reference structure ${\left|E_{ref,k}\right|}^{2}$ to calculate the enhancement in fractional radiative LDOS, and thus the PLE.

In our recent work, we discovered that the nearfield confinement of a resonance can be calibrated by the absorptive decay rate contributed by dye Γ_abs,dye_, where the nearfield region of interest is doped with a dye that absorbs at the resonant wavelength [29], [38]. The absorptive decay rate Γ_abs_ is defined through the power absorbed $P_{\text{abs}}=\Gamma_{\text{abs}}{\left|a\left(\omega_{0}\right)\right|}^{2}$ , where $a\left(\omega_{0}\right)$ is the mode amplitude in CMT and ${\left|a\left(\omega_{0}\right)\right|}^{2}$ is normalized to the total optical energy stored in the mode at resonant frequency *ω*_0_. The contribution of dye can then be identified by considering the power absorbed by the dye medium $P_{\text{abs,dye}}=\Gamma_{\text{abs,dye}}{\left|a\left(\omega_{0}\right)\right|}^{2}$ . We can express *P*_abs,dye_ as the average dissipative energy density of the nearfield in the dye medium at *ω*_0_, giving us:

(2) $$\Gamma_{\text{abs,dye}}=\frac{\int_{\text{dye}}\frac{1}{2}\omega_{0}\varepsilon_{0}\varepsilon^{\prime \prime }\left(r,\omega_{0}\right){\left|E\left(r,\omega_{0}\right)\right|}^{2}{\text{d}}^{3}r}{{\left|a\left(\omega_{0}\right)\right|}^{2}}$$

where *ɛ*_0_ is the permittivity of vacuum and *ɛ*″ is the imaginary part of the relative permittivity [39], [40]. Under the assumption that the dye medium is homogeneous, we can treat $\varepsilon^{\prime \prime }\left(r,\omega_{0}\right)$ as a constant and factor it out of the integral, giving:

(3) $$\int_{\text{dye}}{\left|E\left(r,\omega_{0}\right)\right|}^{2}{\text{d}}^{3}r=\frac{2{\left|a\left(\omega_{0}\right)\right|}^{2}\Gamma_{\text{abs,dye}}}{\omega_{0}\varepsilon_{0}\varepsilon_{\text{dye}}^{\prime \prime }\left(\omega_{0}\right)}$$

which corresponds to the numerator in Eq. (1). Based on CMT, the steady-state solution gives ${\left|a\left(\omega \right)\right|}^{2}=\frac{\left(\Gamma_{\text{rad}}/2\right)\sqrt{1-A_{0}}{\left|\left\langle v^{*}|s_{+,k}\right\rangle \right|}^{2}}{{\left(\omega -\omega_{0}\right)}^{2}+{\left(\Gamma_{\text{tot}}/2\right)}^{2}}$ , where $\left|v\right\rangle$ is the in-coupling constant and $\left|s_{+,k}\right\rangle$ is the incident wave vector for the incident *ℏ* **k** in CMT [41]. Γ_tot_ = Γ_rad_ + Γ_abs,dye_ + Γ_abs,MS_ is the total decay rate, Γ_rad_ is the radiative decay rate and Γ_abs,MS_ is the absorptive decay rate contributed by the metasurface (excluding the dye medium). Since we are primarily dealing with cases where the non-resonant absorptivity *A*_0_ is zero at the emission wavelength, the $\sqrt{1-A_{0}}$ term equals 1.

For simplicity, we choose a reference that is a uniform thin film of the same dye-containing homogeneous medium, placed on the same substrate without the metasurface. Thus, we get a planar structure in which determining $E_{\text{ref},k}\left(r,\omega \right)$ becomes straightforward and we get $\left\langle s_{+,k}|s_{+,k}\right\rangle =\frac{1}{2}\sqrt{\frac{\varepsilon_{0}\varepsilon }{\mu_{0}\mu }}{\left|E_{\text{ref},k}\right|}^{2}\times \left(\text{unit}\;\text{area}\right)$ . Combining the above equations gives:

(4) $$\text{PL}{\text{E}}_{-k}\left(\omega_{0}\right)=\frac{c\Gamma_{\text{rad}}}{\omega_{0}t{\Gamma_{\text{tot}}}^{2}}\frac{\Gamma_{\text{abs},\text{dye}}}{\kappa }\frac{{\left|\left\langle v^{*}|s_{+,k}\right\rangle \right|}^{2}}{\left\langle s_{+,k}|s_{+,k}\right\rangle }$$

where $t=\int_{\text{dye}}1/\left(\text{unit}\;\text{area}\right){\text{d}}^{3}r$ is the thickness of the reference dye medium, *κ* is the extinction coefficient of the dye medium and *c* is the speed of light in vacuum. We can slightly simplify Eq. (4) by defining the radiative decay rate associated with the incident *ℏ* **k** (and polarization **q**) as $\Gamma_{\text{rad},\;\text{in}}=\Gamma_{\text{rad}}\frac{{\left|\left\langle v^{*}|s_{+,k}\right\rangle \right|}^{2}}{\left\langle s_{+,k}|s_{+,k}\right\rangle }=\Gamma_{\text{rad}}\left|v_{k}^{2}\right|$ and dropping the subscript −**k**, the PLE thus can be expressed as:

(5) $$\text{PLE}\left(\omega_{0}\right)=\frac{c\Gamma_{\text{rad},\;\text{in}}}{\omega_{0}t{\Gamma_{\text{tot}}}^{2}}\frac{\Gamma_{\text{abs},\text{dye}}}{\kappa }$$

## 3 Results and discussion

The influence of different factors on the PLE can be dissected by re-arranging Eq. (5) into $\text{PLE}\left(\omega_{0}\right)=\left(\frac{\omega_{0}}{\Gamma_{\text{tot}}}\right)\left(\frac{\Gamma_{\text{abs},\text{dye}}}{\kappa t}\right)\left(\frac{\Gamma_{\text{rad},\;\text{in}}}{\Gamma_{\text{tot}}}\right)\left(\frac{c}{\omega_{0}^{2}}\right)$ . The first term *ω*_0_/Γ_tot_ is the *Q* factor. The second term $\Gamma_{\text{abs},\text{dye}}/\left(\kappa t\right)$ is related to *V*. As discussed in [29], $\Gamma_{\text{abs},\text{dye}}/\left(\kappa t\right)$ is proportional to the averaged 1/*V* over the dye medium in which the photoluminescence process occurs. The third term Γ_rad,in_/Γ_tot_ describes the out-coupling efficiency of the optical cavity, which is an influential factor when analyzing the PLE of *q*-BIC, yet was not captured in the classical representation of the Purcell factor.

### 3.1 Lossless *q*-BIC on TiO_2_ metasurface

**Figure 2:**
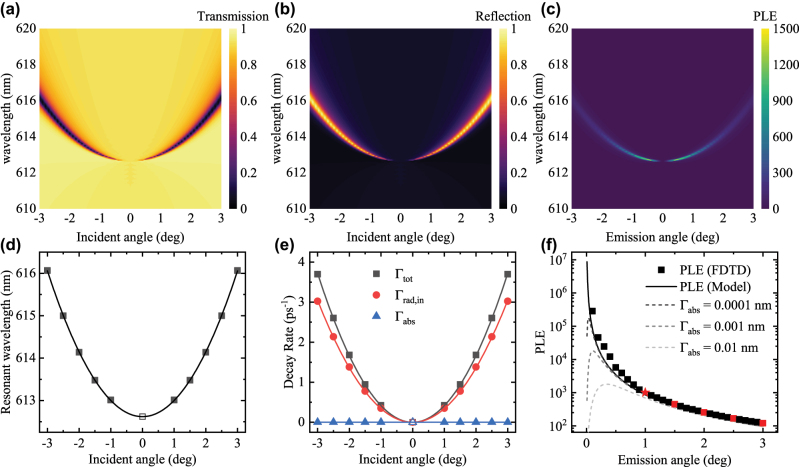
The numerically simulated (a) transmissivity, (b) reflectivity and (c) PLE of the lossless TiO_2_ metasurface. The (d) resonant wavelength and (e) decay rates Γ_tot_, Γ_rad,in_ and Γ_abs_ are plotted as a function of the incident angle *θ*. (f) The numerical PLE and predicted PLE are plotted as a function of the emission angle *θ*. The PLE predicted with a small artificial Γ_abs_ are plotted as dashed lines.

We can first get a basic understanding of the behavior of the PLE of *q*-BIC by considering an ideal, lossless metasurface that supports a symmetry-protected BIC. The detailed design of the metasurface is described in the supplementary materials [41]. The angle-dependent transmissivity *T* and reflectivity *R* of the metasurface were numerically simulated and plotted in Figure 2(a) and (b). We can clearly observe the symmetry-protected BIC at incident angle *θ* = 0° and wavelength *λ* = 612.6 nm, and as detuning *θ* was introduced, the *q*-BIC emerges with a ±*θ* symmetry. The nearfield in the dye medium was also recorded and the numerically predicted PLE was calculated directly by Eq. (1) and plotted in Figure 2(c). The *T* and *R* of each incident angle were fitted to obtain the spectral parameters *ω*_0_, Γ_tot_ and Γ_rad,in_, with Eq. (S4) and (S5), respectively [41]. The extinction coefficient *κ* of the dye medium was modulated to calibrate the factor Γ_abs,dye_/*κ*, which is the slope of Γ_abs_ against *κ* [41].

Since we focus on analyzing the behavior of the metasurface within a small detuning from the BIC, we can model the change in spectral parameters by a polynomial expansion, specifically up to the quadratic term. The *ω*_0_ and the decay rates are plotted in Figure 2(d) and (e) with their polynomial fit. As illustrated in Figure 2(e), Γ_abs_ remains zero for all incident angles while Γ_tot_ and Γ_rad,in_ increases quadratically from the BIC point. On the other hand, we can see that the factor Γ_abs,dye_/*κ* remain mostly constant throughout the fitted range with a slight increase at larger *θ* (see Fig. S1(f)), which indicates similar nearfield confinement at different *θ*. As a result, the total *Q* factor diverges at the BIC and decreases following the inverse-squared law of the detuning (*θ*) [42], [43], [44]. We then predict the PLE with the best fit of the parameters and compare with the numerical PLE in Figure 2(f). The PLE increases rapidly as we approach the BIC and appears to diverge at the BIC. Since non-radiative decay rates remain zero while Γ_tot_ and Γ_rad,in_ both varies quadratically against the incident angle, the out-coupling efficiency Γ_rad,in_/Γ_tot_ remains constant over all incident angles and did not influence (or reduce) the PLE.

However, as illustrated in Figure 2(f), the behavior is significantly different once we artificially introduce a small Γ_abs_, which models a non-zero absorption loss and/or random scattering loss. Since the Γ_abs_ increases Γ_tot_ slightly while not affecting Γ_rad,in_, the out-coupling efficiency Γ_rad,in_/Γ_tot_ is no longer a constant but drops sharply around the BIC, influencing the PLE to hit a maximum and drop sharply around the BIC. We can see this effect even with very small artificial Γ_abs_ equivalent to a linewidth broadening of 0.0001 nm, which is difficult to observe with conventional spectrometers. This prompts us to explore in more detail the effect of absorption and random scattering loss.

### 3.2 Lossy *q*-BIC on Si metasurface

**Figure 3:**
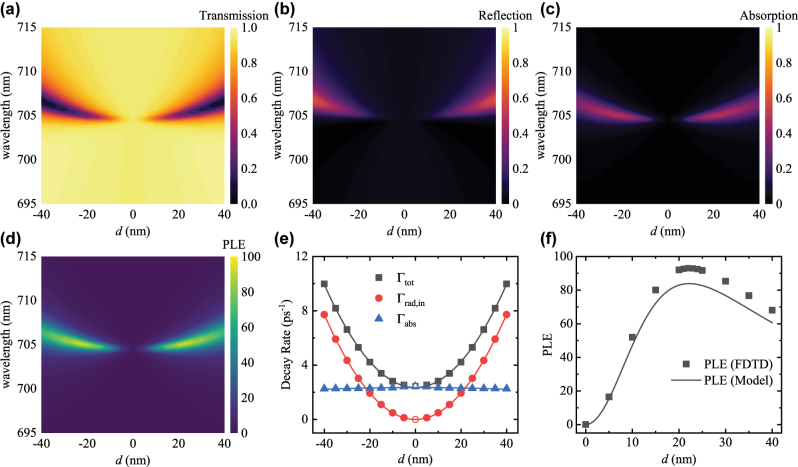
The numerically simulated (a) transmissivity, (b) reflectivity, (c) absorptivity and (d) PLE of the bipartite Si metasurface. (e) The decay rates Γ_tot_, Γ_rad,in_ and Γ_abs_ are plotted as a function of the detuning *d*. (f) The numerical PLE and predicted PLE are plotted as a function of the detuning *d*.

In the previous section, we revealed that even a very small absorption loss from the metasurface would inevitably change the characteristic behavior of PLE from BIC. However, material loss is inevitable in realistic metasurfaces [45]; nanofabrication also introduces inherent roughness into the metasurface structure, which introduces random scattering loss in application [46]. This prompts us to take a closer look into BIC supported on realistic materials with loss instead of an ideal, lossless model. Therefore, we consider a symmetry-protected BIC supported on a bipartite Si metasurface, as described in the supplementary materials [41]. The symmetry-protected BIC at *λ* = 704.5 nm was detuned by displacing one of the Si nanoparticles by *d*, and the *d*-dependent transmissivity *T*, reflectivity *R*, absorptivity *A* = 1 − *T* − *R* and PLE were numerically simulated and plotted in Figure 3(a)–(d). The *T*, *R* and *A* were fitted to obtain the spectral parameters *ω*_0_, Γ_tot_ and Γ_rad,in_, while Γ_abs,dye_/*κ* was fitted by modulating *κ* of the dye medium [41].

As shown in Figure 3(e), Γ_abs_ remains almost constant against *d* with a small decrease at increasing *d*. This decrease is due to the gradual redshift of *ω*_0_ as *d* increases and Si being less absorptive at longer wavelengths. On the other hand, Γ_rad,in_ = Γ_rad_ is zero at the BIC and increases quadratically from there. This is like what we observed in the lossless case, and we expect a quadratic dependency due to the limited range and the symmetry about ±*d*. Therefore, Γ_tot_, the sum of Γ_abs_ and Γ_rad_, roughly follows the same shape of Γ_rad_ but is displaced upwards by Γ_abs_. Since *d* only shifts the position of the Si nanoparticles and does not increase or reduce the amount of dye medium, the factor Γ_abs,dye_/*κ* also remains mostly constant against *d*, as illustrated in Fig. S2(e) [41]. This allows us to assume Γ_abs,dye_/*κ* is constant and use the value at *d* = 20 nm for the prediction of PLE. As illustrated in Figure 3(f), the numerically simulated PLE aligned with the PLE predicted by Eq. (5). First, the PLE increases when reducing *d* and approaching the BIC due to the increase in *Q* factor. However, as we get closer to the BIC configuration, the decrease in the out-coupling efficiency overwhelms the improvement in *Q* factor and the PLE drops sharply near the BIC.

Interestingly, the maximum in PLE occurs very close to the point at which Γ_abs_ and Γ_rad_ intersects. This coincides with the critical coupling condition, which states that light–matter interaction is maximized when Γ_abs_ = Γ_rad_ [32]. This also presents a new perspective on how to optimize Purcell enhancement with realistic metasurfaces. First, Γ_abs_ should be as small as possible, which can be achieved by using low-loss materials for the metasurface [45], or reducing plasmonic loss with nonlocal resonances [43], [47]. The Γ_abs_ sets a lower limit to Γ_tot_ and thereby also imposes an upper bound to the *Q* factor. Then, Γ_rad_ should be tuned to equal Γ_abs_ to achieve the critical coupling condition and maximize light–matter interaction. If Γ_rad_ is too small, the system is dominated by the absorptive loss and most of the enhanced emission would be absorbed by the metasurface instead of out-coupled. While for large Γ_rad_, the increase in out-coupling efficiency is not worth the decrease in *Q* factor and therefore the PLE is reduced.

### 3.3 Asymmetric TiO_2_ metasurface supporting accidental *q*-BIC

**Figure 4:**
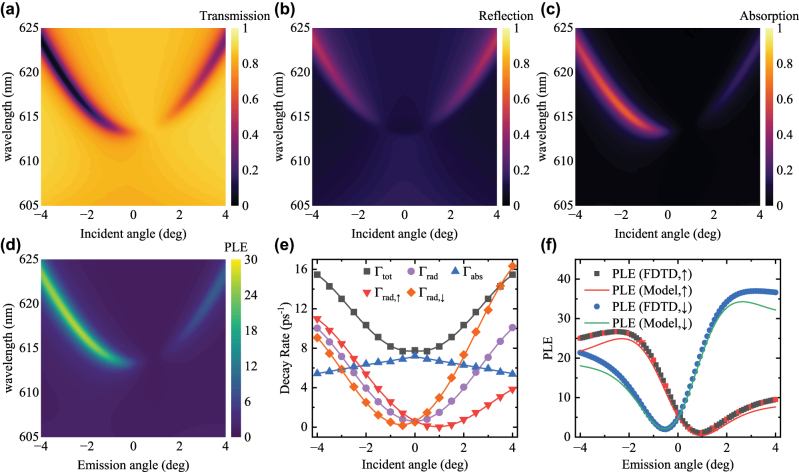
The numerically simulated (a) transmissivity, (b) reflectivity, (c) absorptivity and (d) PLE of the asymmetric TiO_2_ metasurface. (e) The decay rates Γ_tot_, Γ_rad_, Γ_abs_, Γ_rad,↑_ and Γ_rad,↓_ are plotted as a function of the incident angle *θ*. (f) The numerical PLE and predicted PLE are plotted as a function of the incident angle *θ*.

In the following sections, we explore the behavior of an off-Γ accidental *q*-BIC on experimental and simulated metasurfaces. We fabricated asymmetric TiO_2_ metasurfaces that support accidental *q*-BICs by glancing angle deposition of Ti followed by rapid thermal annealing (RTA) on symmetric TiO_2_ metasurfaces [41]. A numerical model is built to replicate the behavior of the experimental asymmetric TiO_2_ metasurface, which the design is described in the supplementary materials [41]. Since the TiO_2_ was formed through RTA of Ti, we expect some roughness in the metasurface and the random scattering loss is modelled by adding an extinction coefficient $\kappa_{\text{Ti}{\text{O}}_{2}}$ = 0.005 to the TiO_2_ material model. While the numerical simulation only simulates the coherent and periodic part of the EM field, the randomly scattered photons are decoherent from the resonant field. Therefore, we choose to model the random scattering loss as absorption as both absorbed photons and randomly scattered photons would not be part of the simulated field. We also assumed a uniform scattering density within the TiO_2_ material, which is intended to account for both the surface roughness of the material as well as structural inhomogeneity within the material. As we can see in the angle-dependent *T*, *R*, *A* and PLE plotted in Figure 4(a)–(d), an accidental BIC is observed at an oblique incident angle *θ* = 0.8° and *λ* = 614 nm. The *T*, *R* and *A* were fitted to obtain the spectral parameters *ω*_0_, Γ_tot_ and Γ_rad,in_, and Γ_abs,dye_/*κ* was also fitted [41].

The decay rates of the accidental *q*-BIC on the asymmetric TiO_2_ metasurface behave markedly differently from that of the symmetric cases. As illustrated in Figure 4(e), Γ_abs_ is largest at normal incident and decreases following a linear style when increasing |*θ*|. The Γ_rad_ is also symmetrical over ±*θ* and remains non-zero at all *θ*. On the other hand, the asymmetry in the metasurface splits the point where Γ_rad,↑_ and Γ_rad,↓_ touch zero, corresponding to the ports at the superstrate (↑) and substrate (↓) sides, towards positive and negative incident angles respectively. Γ_rad,↑_ and Γ_rad,↓_ now touch zero at 0.92° and −0.53° respectively. To use the parameters to predict the PLE, the decay rates were fitted with fourth-order polynomials. The polynomial fits of the parameters were then substituted into Eq. (5) to derive a prediction of the PLE. As illustrated in Figure 4(f), Eq. (5) predicted the numerical PLE with good accuracy (see Fig. S3(e) for the simulated substrate side PLE) [41]. The PLE at the superstrate side shows a minimum at 0.9° while that at the substrate side shows a minimum at −0.5°. A local maximum in PLE is observed at −2.5° at the superstrate side and the model predicted it at −2.2°. On the substrate side, a local maximum is observed at 3.2° followed by a very slow decrease in PLE, while the model predicts a local maximum at 2.5°. Overall, we see larger discrepancies between the analytical and numerical PLE near the end of the fitted range. Due to the nature of polynomial regression, the edge of the data is usually less well represented compared to the data near the center (normal incident), as the higher-order terms are truncated and they are only significant for larger |*θ*|. Therefore, in the trade-off between overfitting and poor fitting accuracy, we proceeded with fourth-order polynomials as they fitted the decay rates better when compared to second-order polynomials, while further increasing the polynomial order did not improve significantly upon that. While this choice is unlikely to lead us to overfitting, this also limits our accuracy, especially for the data near the edges of the fitted range.

It is worth noting that *R* remained symmetric over ±*θ*, even on an asymmetric structure. We were also able to predict the PLE at both the superstrate side and the substrate side, only with the spectra incident from the superstrate side. Both features are because Lorentz reciprocity ensures the in-coupling and out-coupling process are symmetric, which ensures a symmetric *R*. Also, by simultaneously fitting *T* and *R* of ±*θ*, we were able to determine the in-coupling constants of all ports and thus model the PLE at both the superstrate side and the substrate side.

### 3.4 Asymmetric TiO_2_ metasurface fabricated by glancing angle deposition

**Figure 5:**
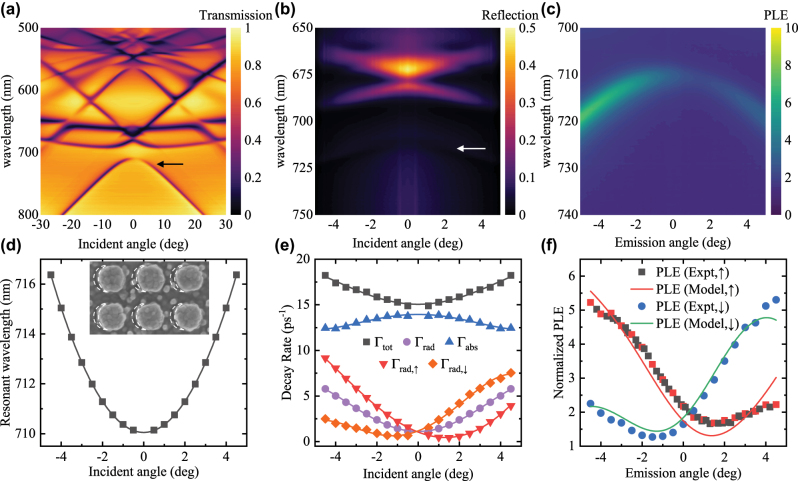
The measured (a) transmissivity, (b) reflectivity, and (c) PLE (*I*/*I*_0_) of the asymmetric TiO_2_ metasurface fabricated by glancing angle deposition. The arrows indicate the mode of interest. The (d) resonant wavelength and (e) decay rates Γ_tot_, Γ_rad_, Γ_abs_, Γ_rad,↑_ and Γ_rad,↓_ are plotted as a function of the incident angle *θ*. The inset in (d) highlights the asymmetry in the TiO_2_ metasurface. The void formed under the shadow of TiO_2_ nanoparticles during glancing angle deposition are outlined by the dashed lines. (f) The re-normalized PLE and predicted PLE are plotted as a function of the emission angle *θ*.

In this section, we experimentally investigate the behavior of accidental *q*-BIC on the asymmetric TiO_2_ metasurfaces. The asymmetric metasurface was fabricated by glancing angle deposition on a symmetric TiO_2_ metasurface. A Ti thin film (100 nm) was deposited on a SiO_2_ glass substrate by electron beam deposition, and a resist (TU7, Obducat) was spin-coated on top. A resist array with square nanoparticles of sides 160 nm arranged in a square lattice of period 370 nm was then fabricated by nanoimprinting. The pattern was transferred to the Ti thin film by O_2_ ashing (RIE-10NR) and etching with Cl_2_ and CHF_3_ (NLD-570). The Ti metasurface was then oxidized by RTA (900 °C, 10 min) under O_2_ atmosphere to fabricate a symmetric TiO_2_ metasurface. A 20 nm thin film of Ti was deposited onto the TiO_2_ metasurface from a glancing angle by tilting the substrate in an electron beam deposition apparatus. The substrate was tilted by 30° along the *xz*-plane. The newly deposited Ti was also oxidized with the same RTA process to create the asymmetric TiO_2_ metasurface. As highlighted in the SEM image of the asymmetric metasurface shown in the inset of Figure 5(d), voids formed only on the left-hand side of the TiO_2_ nanoparticles, at the shadow of the nanoparticles during glancing angle deposition. Finally, a PMMA layer (450 nm) was spin-coated onto the TiO_2_ metasurface. The PMMA is doped with an organic dye (Lumogen F 305 Red) at 1 wt% (weight percent), and the PLE on this Lumogen dye is evaluated. The *T*, *R* and PLE (*I*/*I*_0_) were measured on the rotation stages illustrated in the supplementary materials [41]. The measured *T*, *R* and PLE (*I*/*I*_0_) are plotted in Figure 5(a)–(c). We focus on the branch showing an accidental BIC at *θ* = 1.2° and *λ* = 710 nm. The spectral parameters *ω*_0_, Γ_tot_ and Γ_rad,in_ were fitted from the measured spectra directly. However, to calibrate the nearfield confinement with Γ_abs,dye_/*κ*, we need to use a dye that absorbs light at around *λ* = 700–800 nm. In place of the PMMA layer doped with Lumogen, we spin-coated a similar PMMA layer with 0.25 wt% of IR780 iodide onto the asymmetric TiO_2_ metasurface. The *T* and *R* of the metasurface with IR780 are then measured to fit for (mainly) Γ_abs_ and thus we derive Γ_abs,dye_/*κ* [41].

The decay rates behaved like the simulated asymmetric TiO_2_ metasurfaces. The Γ_abs_ is largest at normal incident and decreases slightly at larger |*θ*|. As illustrated in Figure 5(e), Γ_rad_ remains symmetric over ±θ, while Γ_rad,↑_ and Γ_rad,↓_ touches zero at 1.3° and −1.2° respectively. The parameters also show a good fit when approximated with fourth-order polynomials. We then use the polynomial fits to derive the predicted PLE through Eq. (5). While our analytical model only predicts enhancement due to the Purcell effect, enhancement in the absorption by the Lumogen dye due to the presence of the metasurface is not described, yet is included in the measured *I*/*I*_0_. To minimize the influence of the absorption enhancement and the uneven excitation profile associated with higher absorption enhancement, the incident angle of the excitation laser was chosen at −31.5°, and the measured PLE was re-normalized against the baseline to isolate the Purcell effect. We can now compare the predicted PLE against the re-normalized PLE. As shown in Figure 5(f), the experimental Purcell enhancement is predicted by our analytical model. We also apply Lorentz reciprocity to predict the backside PLE, which our experimental measurements show good agreement with (see Fig. S4(c) for the measured backside PLE) [41].

## 4 Conclusions

In conclusion, we leveraged the proposed analytical model to analyze the influence of the changes in the out-coupling channels of *q*-BIC on PLE. We first derived the equation that predicts the PLE of *q*-BIC with spectral parameters. Then, we strategically examined several metasurfaces that support *q*-BIC through numerical and experimental analysis to gain a deeper understanding of the PLE. We first investigated a lossless scenario that resembles most closely to an ideal symmetry-protected BIC and examined the basic spectral behavior of *q*-BIC. We then introduced material loss and random scattering loss into the analysis and discussed the effect of these realistic imperfections on the Purcell enhancement. Finally, we also discussed the effects of asymmetry on the spectral properties and PLE of *q*-BIC through systems that support accidental *q*-BIC. We revealed that the interplay between the quality factor and the out-coupling efficiency is the primary contribution towards the PLE of *q*-BIC and maximum enhancement can be achieved by optimizing the detuning from the associated BIC. We also demonstrated the general application methodology with two different dyes for emission measurement and absorption calibration respectively. This calibration approach with two optical materials can be further generalized to analyze different types of luminescent materials, making this analytical approach compatible even with those that do not inherently absorb light. Overall, this work provides an intuitive way to understand the mechanism of photoluminescence enhancement from *q*-BIC, as well as a practical framework for modelling the modal behavior of experimental *q*-BIC directly.

## Supplementary Material

Supplementary Material Details
