# Supplementary material for: Modelling Purcell enhancement of metasurfaces supporting quasi-bound states in the continuum
Source: Nanophotonics. 2025 Dec 9;14(27):5407–15. doi: 10.1515/nanoph-2025-0456 (PMC12717941; doi:10.1515/nanoph-2025-0456)
Supplement: Supplementary file 1 — Supplementary Material Details [file j_nanoph-2025-0456_suppl_001.pdf]

# Supplementary Materials: Modelling Purcell enhancement of metasurfaces supporting *quasi*-bound states in the continuum

Joshua T. Y. Tse[1,2]*, Taisuke Enomoto[1], Shunsuke Murai[2], and Katsuhisa Tanaka[1]

[1]Department of Material Chemistry, Graduate School of Engineering, Kyoto University, Katsura, Kyoto 615-8510, Japan

[2]Department of Physics and Electronics, Graduate School of Engineering, Osaka Metropolitan University, Osaka 599-8531, Japan

*Email: jtytse@omu.ac.jp

## S1. MODIFIED TEMPORAL COUPLED-MODE THEORY

We model the spectral behavior of the $q$-BIC modes with a modified CMT, which describes optical resonances as parametric oscillators and condenses the nearfield spatial profile into parameters that influence the spectral response of the resonators.[1-3] The mode amplitude $a$ of the $q$-BIC mode and the outgoing wave $|s_-\rangle$ follow the equations:

$$\frac{\mathrm{d}}{\mathrm{d}t}a(t) = \left(i\omega_0 - \frac{\Gamma_{\mathrm{tot}}}{2}\right)a(t) + \sqrt{\frac{\Gamma_{\mathrm{rad}}}{2}}\sqrt[4]{1-A_0}\langle v^*|s_+\rangle \tag{S1}$$

$$|s_-\rangle = \sqrt{1-A_0}\mathbf{C}|s_+\rangle + a(t)\sqrt{\frac{\Gamma_{\mathrm{rad}}}{2}}\sqrt[4]{1-A_0}|v\rangle \tag{S2}$$

where $\omega_0$ is the resonant frequency, $A_0$ is the non-resonant absorptivity and the total decay rate $\Gamma_{\mathrm{tot}}$ is the sum of the radiative decay rate $\Gamma_{\mathrm{rad}}$ and the absorptive decay rate $\Gamma_{\mathrm{abs}}$ of the $q$-BIC mode. $|s_\pm\rangle = (s_{\pm,\mathrm{TE},\uparrow} \quad s_{\pm,\mathrm{TM},\uparrow} \quad s_{\pm,\mathrm{TE},\downarrow} \quad s_{\pm,\mathrm{TM},\downarrow})^{\mathrm{T}}$ is the incident(+)/outgoing(–) wave vector normalized to the incident power,

$$\mathbf{C} = \begin{pmatrix} t_{0,\mathrm{TE}} & 0 & r_{0,\mathrm{TE}} & 0 \\ 0 & t_{0,\mathrm{TM}} & 0 & r_{0,\mathrm{TM}} \\ r_{0,\mathrm{TE}} & 0 & t_{0,\mathrm{TE}} & 0 \\ 0 & r_{0,\mathrm{TM}} & 0 & t_{0,\mathrm{TM}} \end{pmatrix}$$

is the direct scattering matrix where $t_{0,\mathrm{TE/TM}}$ and $r_{0,\mathrm{TE/TM}}$ are the non-resonant transmissivity and reflectivity coefficient respectively, and $|v\rangle = (v_{\mathrm{TE},\uparrow} \quad v_{\mathrm{TM},\uparrow} \quad v_{\mathrm{TE},\downarrow} \quad v_{\mathrm{TM},\downarrow})^{\mathrm{T}}$ is the in-coupling constant normalized by $\langle v|v\rangle = 2$. The subscript TE/TM denotes the polarization and $\uparrow/\downarrow$ indicates the upwards/downwards ports at the superstrate/substrate sides respectively. We can solve for the steady-state solution of the modified CMT by considering solutions of form $a(t) = a(\omega)e^{i\omega t}$, which gives:

$$i\omega a(\omega) = \left(i\omega_0 - \frac{\Gamma_{\mathrm{tot}}}{2}\right)a(\omega) + \sqrt{\frac{\Gamma_{\mathrm{rad}}}{2}}\sqrt[4]{1 - A_0}\langle v^*|s_+\rangle \tag{S3}$$

from Eq. (S1) and can be simplified to $a(\omega) = \frac{\sqrt{\Gamma_{\mathrm{rad}}/2}\sqrt[4]{1-A_0}\langle v^*|s_+\rangle}{i(\omega-\omega_0)+\Gamma_{\mathrm{tot}}/2}$ or $|a(\omega)|^2 = \frac{(\Gamma_{\mathrm{rad}}/2)\sqrt{1-A_0}|\langle v^*|s_+\rangle|^2}{(\omega-\omega_0)^2+(\Gamma_{\mathrm{tot}}/2)^2}$. Then, we can use the steady-state solution to derive the (TE-) transmissivity and reflectivity with incident from the superstrate side to be:

$$T_{\mathrm{TE}} = (1 - A_0)\left|t_{0,\mathrm{TE}} + \frac{\Gamma_{\mathrm{rad}}}{2}\frac{v_{\mathrm{TE},\uparrow(\mathrm{in})}v_{\mathrm{TE},\downarrow(\mathrm{out})}}{i(\omega - \omega_0) + \Gamma_{\mathrm{tot}}/2}\right|^2 \tag{S4}$$

$$R_{\mathrm{TE}} = (1 - A_0)\left|r_{0,\mathrm{TE}} + \frac{\Gamma_{\mathrm{rad}}}{2}\frac{v_{\mathrm{TE},\uparrow(\mathrm{in})}v_{\mathrm{TE},\uparrow(\mathrm{out})}}{i(\omega - \omega_0) + \Gamma_{\mathrm{tot}}/2}\right|^2 \tag{S5}$$

The subscript in/out denotes whether the coupling constant is associated with the incident or the outgoing port. This distinction is helpful when dealing with oblique incidents on asymmetric metasurfaces as they use different ports (with different coupling constants) for in-coupling and out-coupling. The absorptivity under the same incident can also be derived by considering $A = 1 - \frac{\langle s_-|s_-\rangle}{\langle s_+|s_+\rangle}$, which we find:

$$A_{\mathrm{TE}} = A_0 + (1 - A_0)\frac{\Gamma_{\mathrm{abs}}\Gamma_{\mathrm{rad}}}{2}\frac{\left|v_{\mathrm{TE},\uparrow(\mathrm{in})}^2\right|}{(\omega - \omega_0)^2 + (\Gamma_{\mathrm{tot}}/2)^2} \tag{S6}$$

where $\Gamma_{\mathrm{rad}}\left|v_{\mathrm{TE},\uparrow(\mathrm{in})}^2\right| = \Gamma_{\mathrm{rad}}\frac{|\langle v^*|s_+\rangle|^2}{\langle s_+|s_+\rangle} \equiv \Gamma_{\mathrm{rad,in}}$. Similar results can also be derived for the TM counterpart, where the subscripts TE are replaced by TM. More importantly, the spectral parameters used in Eq. (5), $\omega_0$, $\Gamma_{\mathrm{tot}}$ and $\Gamma_{\mathrm{rad,in}}$, can be determined by fitting the measured/simulated spectra with Eq. (S4) – (S6).

## S2. NUMERICAL SIMULATION DETAILS

We used finite-difference time-domain method (Ansys Lumerical FDTD) to numerically simulate the properties of metasurfaces that support BIC. In Section III (i), we considered an ideal, lossless metasurface that supports symmetry-protected BIC, as illustrated in the inset of Fig. S1(f). The metasurface consists of $TiO_2$ (refractive index $n$ = 2.4) nanoparticle array placed on a $SiO_2$ ($n$ = 1.46) substrate and covered by a PMMA ($n$ = 1.49) thin film. The $TiO_2$ nanoparticles are frustums with a rectangular base of 240 nm by 200 nm, top of 180 nm by 160 nm and height 160 nm, arranged in a 2D square lattice of period 370 nm. The PMMA thin film is 300 nm thick and acts as the dye medium which the nearfield is integrated to compute the numerical PLE. (An extinction coefficient $\kappa$ is added to the PMMA when fitting for $\Gamma_{\text{abs,dye}}/\kappa$, see Section S3) The $SiO_2$ substrate and the vacuum above the PMMA layer are semi-infinite and extend beyond the simulation domain. The $x$- and $y$-boundaries are terminated with periodic boundary conditions to simulate an infinitely extending metasurface, and the $z$-boundaries are terminated by perfectly matched layers (PML).

In Section III (ii), we numerically simulated the symmetry-protected BIC supported on a bipartite Si metasurface, as illustrated in the inset of Fig. S2(e). The metasurface consists of two Si nanocylinder suspended in an index-matching layer ($n$ = 1.46) of thickness 200 nm, and sandwiched by two semi-infinite $SiO_2$ ($n$ = 1.46) layers. The Si nanocylinders with diameter 130 nm and height 90 nm are arranged in a 2D rectangular lattice of periodicity $P_x$ = 470 nm and $P_y$ = 235 nm. The complex refractive index of Si is extracted from [4]. The nanocylinders are aligned in $y$ and separated by $P_x/2 - d$ in $x$. The center of the index-matching layer is vertically aligned with the Si nanocylinders, and it also acts as the dye medium where the nearfield is integrated to compute the numerical PLE. (An extinction coefficient $\kappa$ is added to the index-matching layer when fitting for $\Gamma_{\text{abs,dye}}/\kappa$.) Like the previous one, the $x$- and $y$-boundaries are terminated with periodic boundary conditions, and the $z$-boundaries are terminated by PMLs.

In Section III (iii), we built a numerical model that replicates the behavior of an asymmetric $TiO_2$ metasurface. The asymmetric $TiO_2$ metasurface we referred to was fabricated following the procedures described in Section III (iv), but with the glancing angle deposition done at 45°. The design of the metasurface is illustrated in Fig. S3(f). The designed metasurface consists of a $TiO_2$ (refractive index $n = 2.4 + 0.005i$) structure placed on a $SiO_2$ ($n$ = 1.46) substrate and covered by a PMMA ($n$ = 1.49) thin film. The $TiO_2$ metasurface consists of a slanted frustum with a triangular top, laying on a film of $TiO_2$ with a hole at the shadow of the nanostructure. The PMMA thin film is up to 300 nm thick and acts as the dye medium which the nearfield is integrated to compute the numerical PLE. (An extinction coefficient $\kappa$ is added to the PMMA when fitting for $\Gamma_{\text{abs,dye}}/\kappa$.) Like in Section

III (i), the $SiO_2$ substrate and the vacuum above the PMMA layer are semi-infinite and extend beyond the simulation domain. The $x$- and $y$-boundaries are also terminated with periodic boundary conditions, and the $z$-boundaries are terminated by PMLs.

## S3. THE NEARFIELD CONFINEMENT FACTOR $\Gamma_{abs,dye}/\kappa$

In Section III (i), we studied an ideal, lossless metasurface that supports symmetry-protected BIC. The nearfield confinement of the $q$-BIC is parametrized by the factor $\Gamma_{abs,dye}/\kappa$ as we introduce an extinction coefficient $\kappa$ into the dye medium. Due to the high $Q$ factor, we use a relatively small $\kappa$ to minimize the disturbance towards the nearfield distribution of the $q$-BIC mode. We numerically simulated the $T$ and $R$ for $\theta$ = 1°, 1.5°, 2°, 2.5°, 3°. $\kappa$ was varied from 0.00002 to 0.0001 for $\theta$ = 1° and from 0.0001 to 0.0005 for the rest. The evolution of $\Gamma_{tot}$, $\Gamma_{rad}$ and $\Gamma_{abs}$ against $\kappa$ are shown in Fig. S1(a) – (e). $\Gamma_{abs,dye}/\kappa$ is extracted from the slope of $\Gamma_{abs}$ against $\kappa$ and is plotted in Fig. S1(f) for different $\theta$.

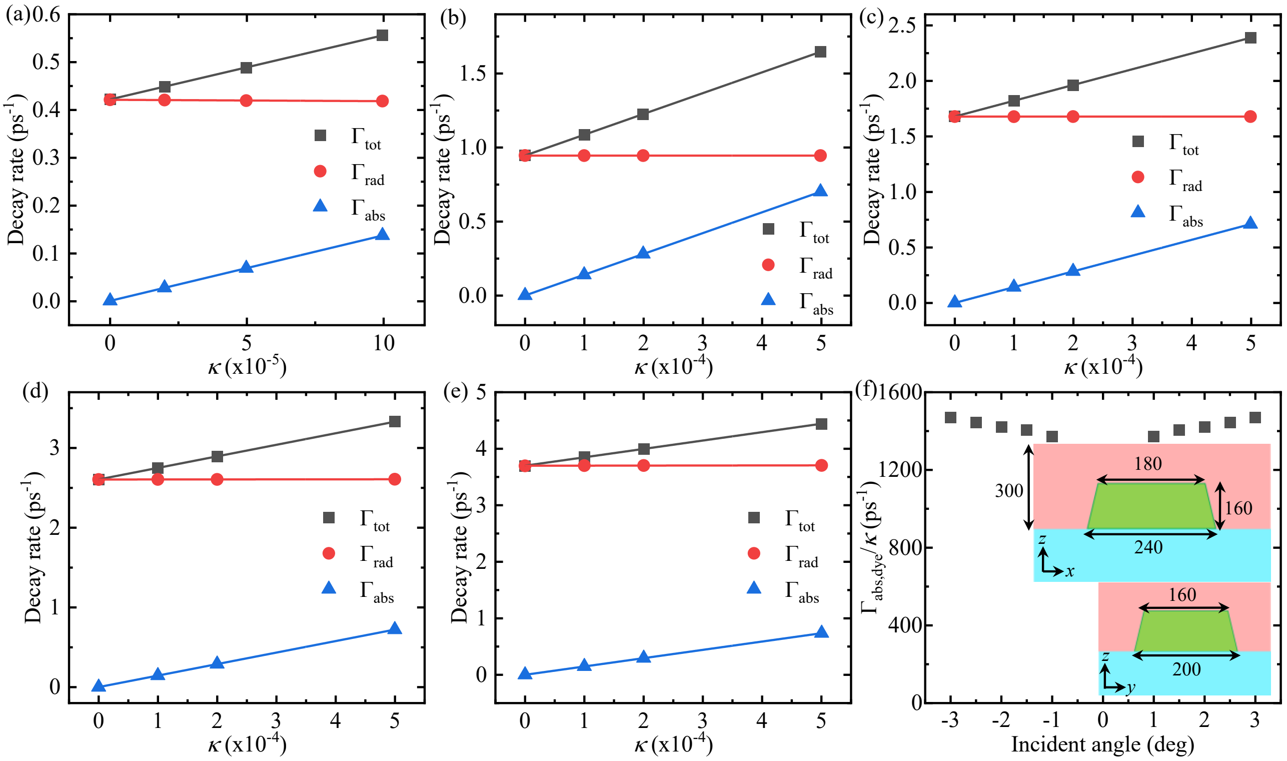


Figure S1. The decay rates $\Gamma_{tot}$, $\Gamma_{rad}$, and $\Gamma_{abs}$ of the lossless $TiO_2$ metasurface at $\theta$ = (a) 1°, (b) 1.5°, (c) 2°, (d) 2.5°, and (e) 3° are plotted as a function of the artificial extinction coefficient $\kappa$ of the dye medium. (f) The resultant $\Gamma_{abs,dye}/\kappa$ is plotted as a function of $\theta$. The inset shows the $xz$- and $yz$-cross section of the metasurface. The pink layer represents the PMMA with dye, the blue layer represents the $SiO_2$ substrate, and the green trapezoid represents the $TiO_2$ nanoparticle. The dimensions are given in nm.

In Section III (ii), we studied a bipartite Si metasurface that supports lossy *q*-BIC. The nearfield confinement of the *q*-BIC is parametrized by the factor $\Gamma_{\mathrm{abs,dye}}/\kappa$ as we introduce an extinction coefficient $\kappa$ into the dye medium. We numerically simulated the *T*, *R* and *A* for $d$ = 10, 20, 30, 40 nm. $\kappa$ was varied from 0.0025 to 0.01. The evolution of $\Gamma_{\mathrm{tot}}$, $\Gamma_{\mathrm{rad}}$ and $\Gamma_{\mathrm{abs}}$ against $\kappa$ are shown in Fig. S2(a) – (d). $\Gamma_{\mathrm{abs,dye}}/\kappa$ is extracted from the slope of $\Gamma_{\mathrm{abs}}$ against $\kappa$ and is plotted in Fig. S2(e) for different *d*.

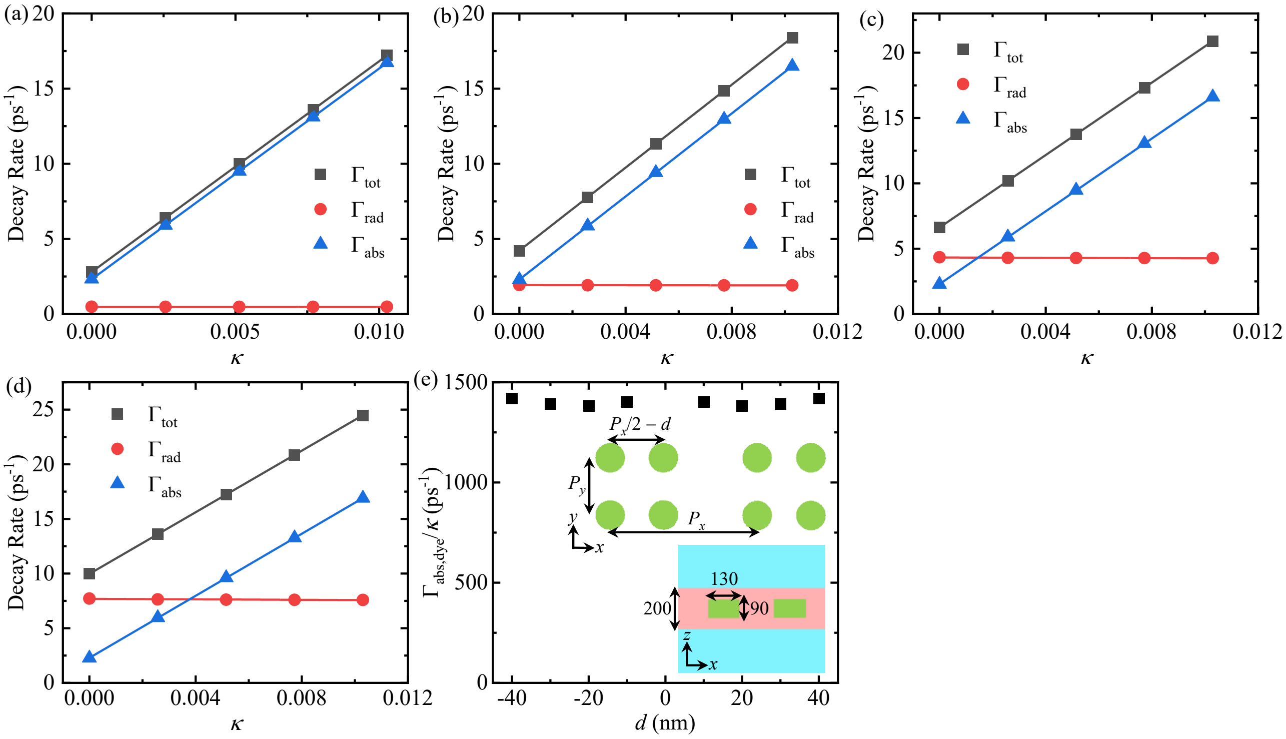


Figure S2. The decay rates $\Gamma_{\mathrm{tot}}$, $\Gamma_{\mathrm{rad}}$, and $\Gamma_{\mathrm{abs}}$ of the Si metasurface at $d$ = (a) 10, (b) 20, (c) 30, and (d) 40 nm are plotted as a function of the artificial extinction coefficient $\kappa$ of the dye medium. (e) The resultant $\Gamma_{\mathrm{abs,dye}}/\kappa$ is plotted as a function of *d*. The inset shows the *xy*- and *xz*-cross section of the Si metasurface. The pink layer represents the index-matching layer with dye, the blue layers represent the $SiO_2$ substrate and superstrate, and the green circles (rectangles) represent the Si nanoparticles. The dimensions are given in nm.

In Section III (iii), we studied an asymmetric $TiO_2$ metasurface that supports accidental *q*-BIC. The nearfield confinement of the *q*-BIC is parametrized by the factor $\Gamma_{\mathrm{abs,dye}}/\kappa$ as we introduce an extinction coefficient $\kappa$ into the dye medium. We numerically simulated the *T*, *R* and *A* for $\theta = \pm 0.5°$, $\pm 1.5°$, $\pm 2.5°$. $\kappa$ was varied from 0.005 to 0.01. The evolution of $\Gamma_{\mathrm{tot}}$, $\Gamma_{\mathrm{rad}}$ and $\Gamma_{\mathrm{abs}}$ against $\kappa$ are shown in Fig. S3(a) – (c). $\Gamma_{\mathrm{abs,dye}}/\kappa$ is extracted from the slope of $\Gamma_{\mathrm{abs}}$ against $\kappa$ and is plotted in Fig. S3(d) for different $\theta$.

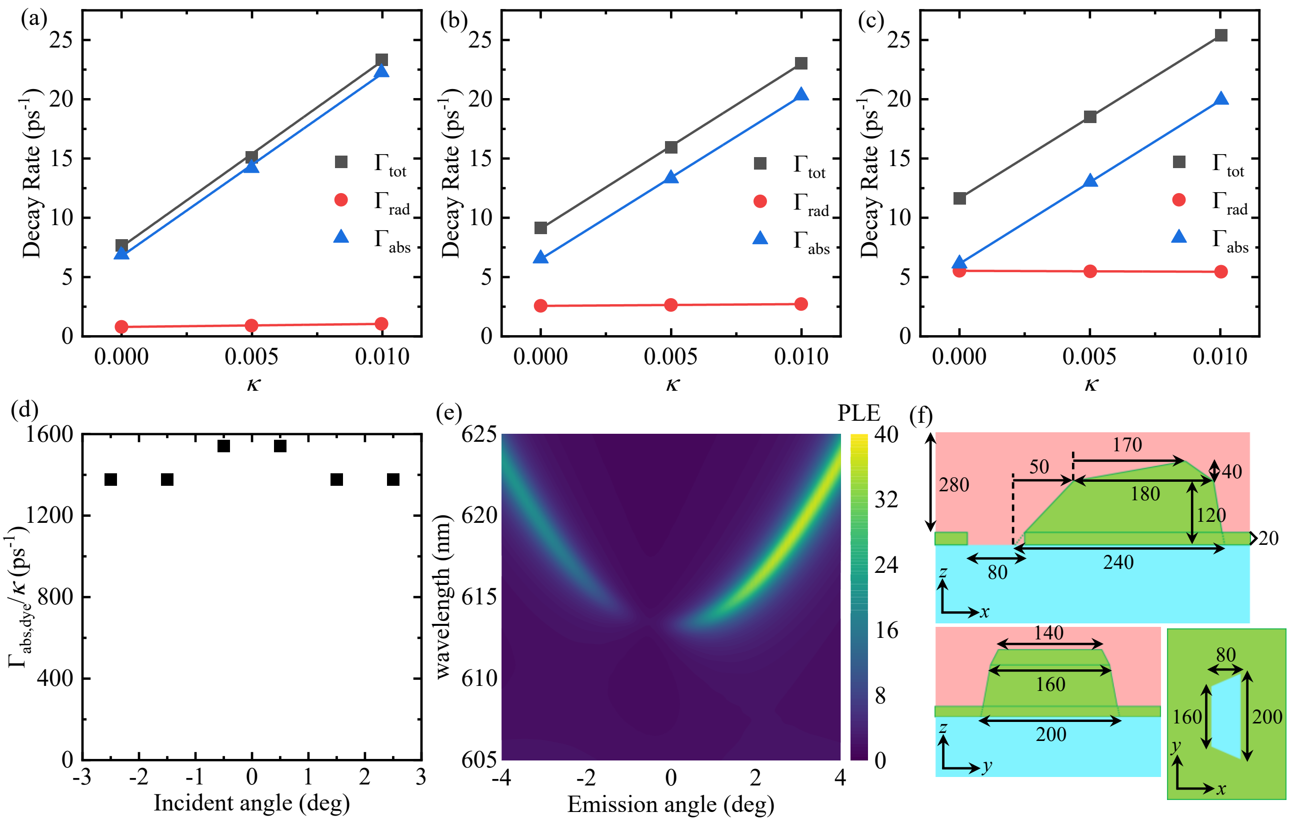


Figure S3. The decay rates $\Gamma_{tot}$, $\Gamma_{rad}$, and $\Gamma_{abs}$ of the asymmetric $TiO_2$ metasurface at $\theta$ = (a) ±0.5°, (b) ±1.5°, and (c) ±2.5° are plotted as a function of the artificial extinction coefficient $\kappa$ of the dye medium. (d) The resultant $\Gamma_{abs,dye}/\kappa$ is plotted as a function of $\theta$. (e) The numerically simulated substrate side PLE of the asymmetric $TiO_2$ metasurface. (f) The *xz*- and *yz*-cross section of the asymmetric $TiO_2$ metasurface is illustrated. The *xy*-cross section of the hole in the $TiO_2$ is also illustrated. The pink layer represents the PMMA with dye, the blue layer represents the $SiO_2$ substrate, and the green parts represent the $TiO_2$ metasurface. The dimensions are given in nm.

In Section III (iv), we experimentally studied an asymmetric $TiO_2$ metasurface that supports accidental *q*-BIC. The nearfield confinement of the *q*-BIC is parametrized by the factor $\Gamma_{abs,dye}/\kappa$ as we introduce the IR780 iodide dye into the PMMA layer. A PMMA layer doped with 0.25 wt.% of IR780 iodide was spin-coated on the asymmetric $TiO_2$ metasurface in place of the PMMA layer doped with the Lumogen dye. The *T* and *R* are measured and plotted in Fig. S4(a) and S4(b). The *T* and *R* are fitted for $\theta$ = ±1°, ±2°, ±3°, ±4°, and the evolution of $\Gamma_{abs}$ against $\kappa$ is shown in Fig. S4(d). (The value of $\kappa$ is discussed in Section S5.) $\Gamma_{abs,dye}/\kappa$ is extracted from the slope of $\Gamma_{abs}$ against $\kappa$.

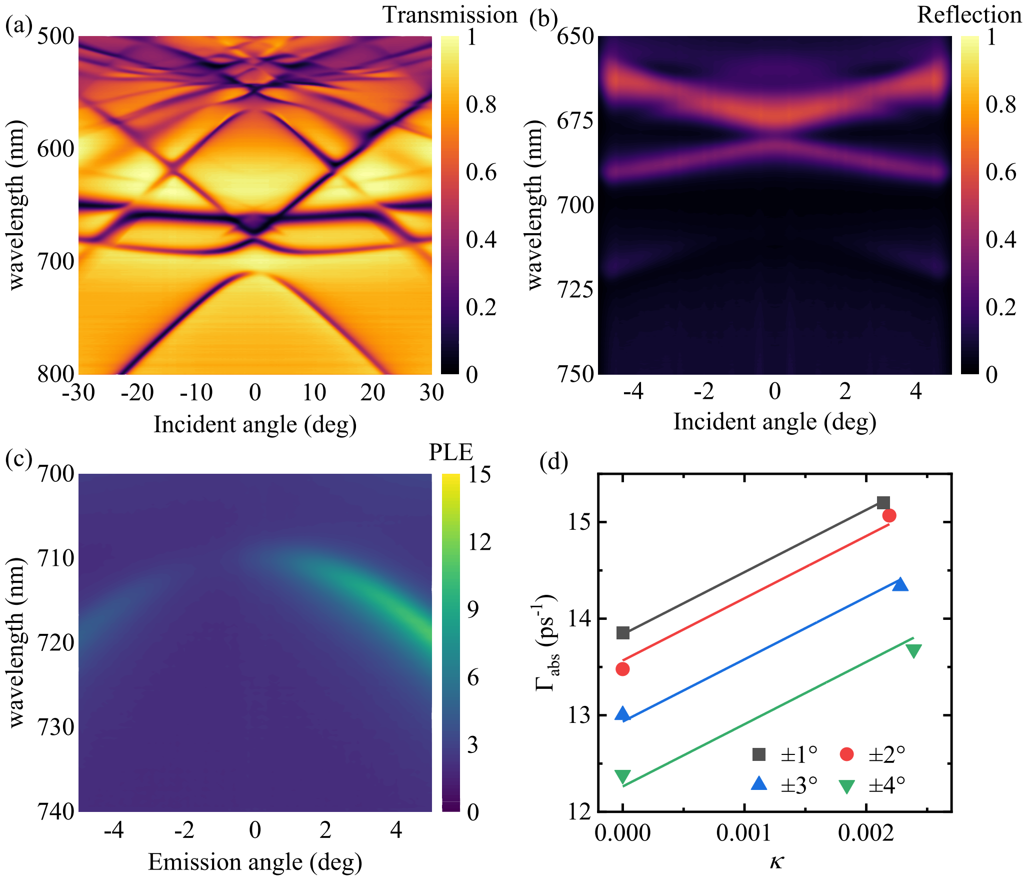


Figure S4. The measured (a) transmissivity and (b) reflectivity of the asymmetric $TiO_2$ metasurface spin-coated with IR780-containing PMMA. (c) The measured substrate side PLE ($I/I_0$) of the asymmetric $TiO_2$ metasurface (with Lumogen). (d) The $\Gamma_{abs}$ at $\theta = \pm1°, \pm2°, \pm3°, \pm4°$ are plotted as a function of $\kappa$.

## S4. OPTICAL MEASUREMENT SETUPS

The *T*, *R* and PLE of metasurfaces are measured optically. The schematic of the measurement setup for transmissivity measurements is shown in Fig. S5(a). A stabilized Tungsten-Halogen lamp (Thorlabs SLS201L) is used as a white light source. The light is coupled through a multimode fiber (Thorlabs M20L01) and collimated with a 5X microscope objective (Nikon MUE42050). The polarization of the incident light is controlled by a polarizer (Thorlabs WP25M-VIS). The light is then directed to the metasurface sample from the PMMA (superstrate) side. The sample is placed on a rotation stage, and the incident angle is controlled by rotating the sample. The transmitted light is collected through a lens (Thorlabs LB1901) into a multimode fiber (Ocean Optics P400-2-UV-VIS) connected to a spectrometer (Ocean Optics FLAME-S).

The schematic of the measurement setup for reflectivity measurements is shown in Fig. S5(b). A Tungsten-Halogen lamp (Ocean Optics HL-2000) is used as a white light source. The light is coupled through a multimode fiber (Ocean Optics QP400-2-UV-VIS) and collimated with a 5X microscope

objective (Nikon MUE42050). The polarization of the incident light is controlled by a polarizer (Thorlabs WP25M-VIS). The light is then directed through a cube beamsplitter (Thorlabs CCM1-BS013) to the metasurface sample from the PMMA (superstrate) side. The reflected light is first reflected by the beamsplitter, and then collected through a lens (Thorlabs LB1901) into a multimode fiber (Ocean Optics P400-2-UV-VIS) connected to a spectrometer (Ocean Optics FLAME-S). The incident arm and the detector arm are mounted on rotation stages and rotate in opposite directions to measure the angle-dependent reflectivity. Due to the constraint from the aperture size of the beamsplitter mount, the reflectivity can only be measured within a ~6° window. However, as discussed in Section III (iii), the reflectivity is expected to be symmetric over $\pm\theta$ due to Lorentz reciprocity. Therefore, the range of the reflection setup is adjusted to utilize larger incident angle rather than to be symmetric about normal incident, resulting in a working range of −1° to 5°.

The experimental setup for emission measurement is shown in Fig. S5(c). A 445 nm blue laser (SLOC BLS-445-1000mW) is used as the excitation source of the Lumogen dye from the substrate side. The collimated laser beam is controlled to be *p*-polarized by a Glan-Thompson polarizer (OptoSigma GTPB-08-21SN) and the incident angle is fixed at $\theta_{in} = 31.5°$. The emission polarization is filtered by a linear polarizer (Thorlabs WP25M-VIS) and the emission $I$ at the superstrate side and angle $\theta$ is focused by a lens (Thorlabs LB1901) into an optical fiber (Ocean Optics P400-2-UV-VIS) connected to a spectrometer (Ocean Optics FLAME-S). The detection arm is rotated around the sample to measure the emission at different $\theta$. The emission from the metasurface sample is normalized against the emission intensity of the Lumogen dye on an unstructured layer $I_0$ to obtain the PLE ($I/I_0$).

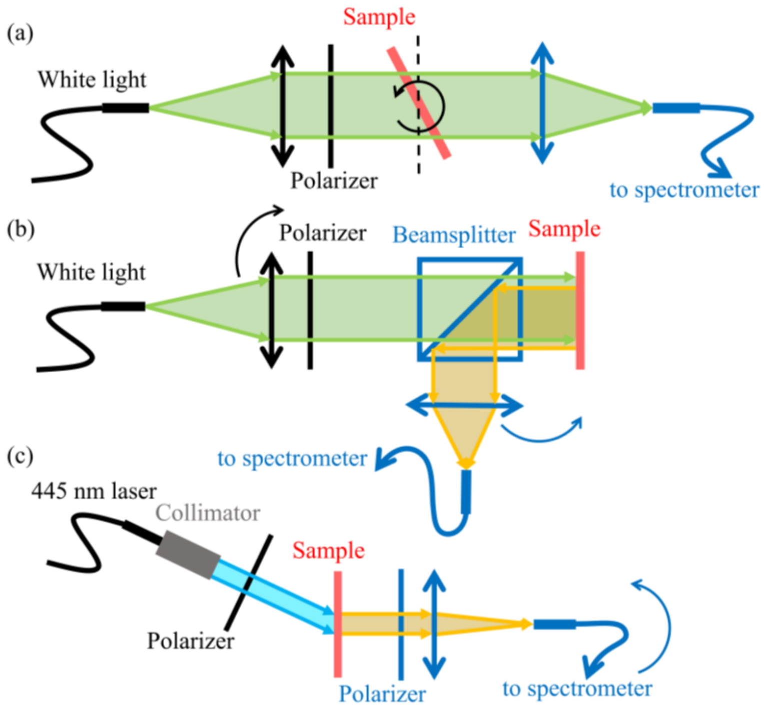

Figure S5. The optical measurement setups for (a) transmission, (b) reflection, and (c) photoluminescence measurements are illustrated. The optical elements of the incident side are drawn in black while that of the detection side are drawn in blue.

## S5. CALIBRATION OF THE ORGANIC DYES

The absorption and emission spectra of Lumogen F 305 red were measured as a reference. A PMMA layer (450 nm) with 1 wt.% of the Lumogen dye was spin-coated on an unstructured $SiO_2$ glass substrate. Fig. S6(a) shows the measured absorption and emission of the Lumogen dye normalized to the respective absorption and emission peak values.

We also measured the extinction coefficient $\kappa$ of the PMMA layer with IR780 iodide. A PMMA layer (450 nm) doped with 0.25 wt.% of IR780 iodide was spin-coated on an unstructured $SiO_2$ glass substrate. The normalized transmissivity of the dye layer was measured by UV/VIS/NIR spectroscopy (JASCO V-770) and was fitted by the Fresnel's Equations. The permittivity of the dye layer was modelled by the generalized Lorentz oscillator model with 6 oscillators.[5] As shown in Fig. S6(b), the best fit is consistent with the measured transmissivity, and the fitted $n$ and $\kappa$ of the dye layer are shown in Fig. S6(c).

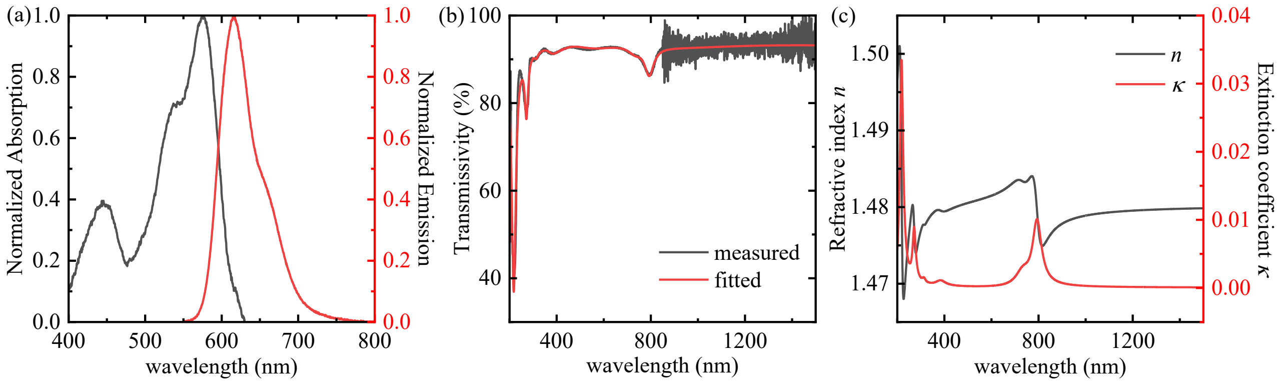


Figure S6. (a) The normalized absorption and emission spectra of Lumogen F 305 red. (b) The measured and fitted transmissivity of the PMMA layer (450 nm) with 0.25 wt.% of IR780 iodide is plotted against the wavelength. (c) The fitted $n$ and $\kappa$ of the PMMA layer with IR780 are plotted against the wavelength.

**References**


1. H. A. Haus, *Waves and Fields in Optoelectronics* (Prentice-Hall, New Jersey, 1984).
2. S. Fan, W. Suh, and J. D. Joannopoulos, Temporal coupled-mode theory for the Fano resonance in optical resonators, J. Opt. Soc. Amer. A **20**, 569 (2003).
3. J. T. Y. Tse, S. Murai, and K. Tanaka, Resonant critical coupling of surface lattice resonances with a fluorescent absorptive thin film, J. Phys. Chem. C **127**, 22212 (2023).
4. E. D. Palik, editor, *Handbook of Optical Constants of Solids*, Vol. 3 (Academic Press, San Diego, 1998).
5. A. F. J. Levi, *Essential Classical Mechanics for Device Physics* (Morgan & Claypool, San Rafael, 2016).
